# 1192. Validation of an Electronic Dashboard in Identifying Excess Duration of Antibiotics for Pneumonia

**DOI:** 10.1093/ofid/ofad500.1032

**Published:** 2023-11-27

**Authors:** Mary Ferranti, Kathleen Degnan, David Do, Keith W Hamilton, Lauren Dutcher

**Affiliations:** Hospital of the University of Pennsylvania, Philadelphia, Pennsylvania; University of Pennsylvania Perelman School of Medicine, Philadelphia, Pennsylvania; Hospital of the University of Pennsylvania, Philadelphia, Pennsylvania; University of Pennsylvania Perelman School of Medicine, Philadelphia, Pennsylvania; University of Pennsylvania Perelman School of Medicine, Philadelphia, Pennsylvania

## Abstract

**Background:**

Many inpatients receive inappropriately prolonged courses of antibiotics for community acquired pneumonia (CAP) and hospital acquired pneumonia (HAP). Identifying these patients is important for use in interventions to reduce unnecessary antibiotic exposure. In this study, we evaluate how well an electronic dashboard identifies inpatients receiving excess treatment for pneumonia and characterize reasons for excess antibiotic duration.

**Methods:**

Within an academic health system, a dashboard was created to generate an alert when inpatients received antibiotics longer than 120 hours (5 days) or azithromycin longer than 72 hours (3 days) for CAP and 168 hours for HAP (7 days). We reviewed a random sample of encounters with an alert between November 2018 and January 2023. Through chart review, we collected medical history, provider documentation of antibiotic indication, duration of treatment, and reasons for excess duration. Descriptive statistics were used to report 1) the proportion of patients with true positive alerts (alerts generated for patients who truly received an excess duration of antibiotics for pneumonia) and false positive alerts (alerts generated but the patients had not received an excess duration of antibiotics for pneumonia) and 2) reasons for true positive and false positive alerts.

**Results:**

Out of 200 patients, 156 (78.0%) were found to have a true positive alert, while 44 (22.0%) had a false positive alert. Of the patients who received a longer duration than needed for pneumonia (i.e. true positives), 24.5% had no documented reason for prolonged duration and 21.5% had documented inadequate clinical improvement as defined in Table 2. Excess days of azithromycin for CAP was also a common reason for true excess duration (13.5%). Incorrect antibiotic indication selection was the most common reason for a false positive alert (31.8%).

**Figure:**
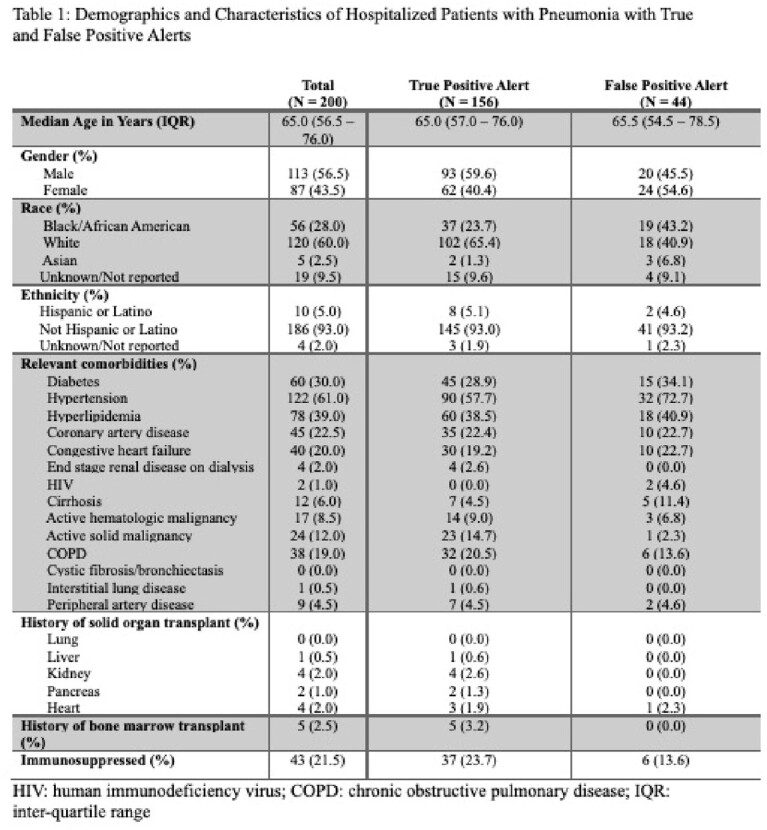

**Figure:**
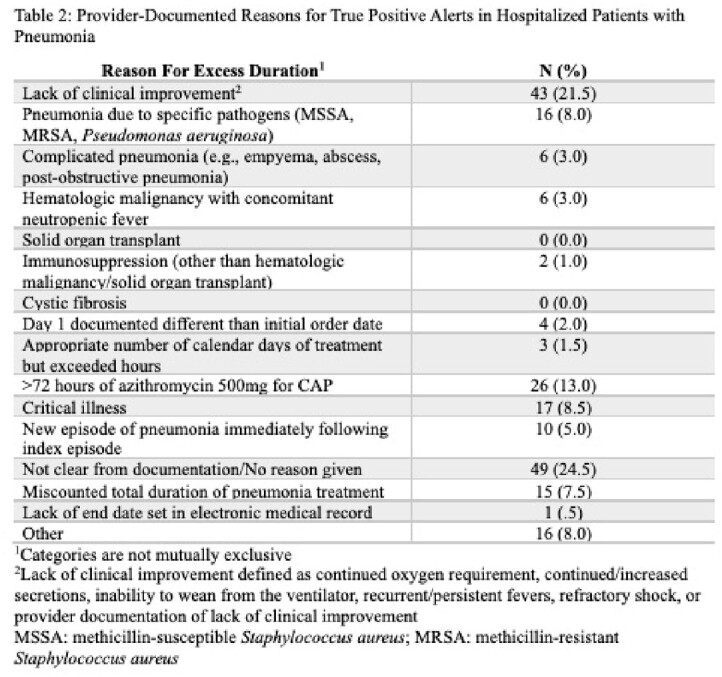

**Figure:**
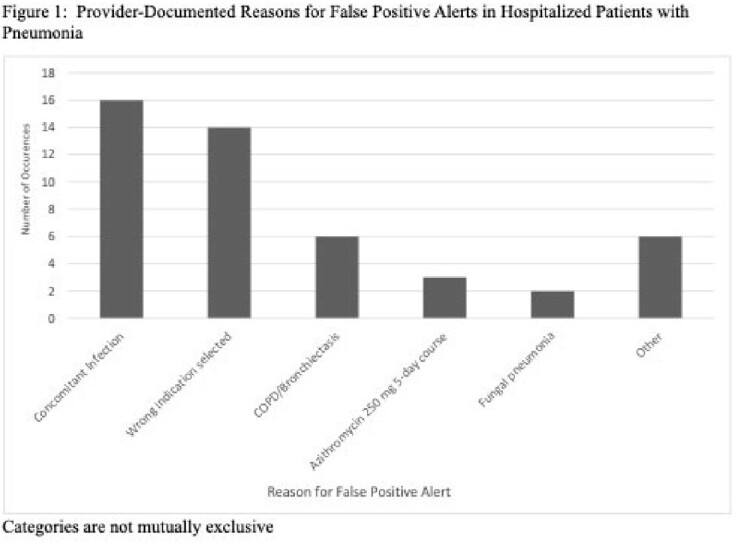

**Conclusion:**

The electronic dashboard is a useful tool that can correctly identify inpatients receiving excess duration of treatment for pneumonia in a majority of cases. Future stewardship endeavors should focus on interventions to reduce such excess duration especially for the most common reasons found.

**Disclosures:**

**Kathleen Degnan, MD**, Gilead: Grant/Research Support

